# Carotenoid Content and Root Color of Cultivated Carrot: A Candidate-Gene Association Study Using an Original Broad Unstructured Population

**DOI:** 10.1371/journal.pone.0116674

**Published:** 2015-01-23

**Authors:** Matthieu Jourdan, Séverine Gagné, Cécile Dubois-Laurent, Mohamed Maghraoui, Sébastien Huet, Anita Suel, Latifa Hamama, Mathilde Briard, Didier Peltier, Emmanuel Geoffriau

**Affiliations:** 1 Agrocampus Ouest, UMR1345 Institut de Recherche en Horticulture et Semences, Angers, France; 2 Université d’Angers, UMR1345 Institut de Recherche en Horticulture et Semences, Angers, France; 3 INRA, UMR1345 Institut de Recherche en Horticulture et Semences, Beaucouzé, France; University of Wisconsin, Food Research Institute, UNITED STATES

## Abstract

Accumulated in large amounts in carrot, carotenoids are an important product quality attribute and therefore a major breeding trait. However, the knowledge of carotenoid accumulation genetic control in this root vegetable is still limited. In order to identify the genetic variants linked to this character, we performed an association mapping study with a candidate gene approach. We developed an original unstructured population with a broad genetic basis to avoid the pitfall of false positive detection due to population stratification. We genotyped 109 SNPs located in 17 candidate genes – mostly carotenoid biosynthesis genes – on 380 individuals, and tested the association with carotenoid contents and color components. Total carotenoids and β-carotene contents were significantly associated with genes zeaxanthin epoxydase (ZEP), phytoene desaturase (PDS) and carotenoid isomerase (CRTISO) while α-carotene was associated with CRTISO and plastid terminal oxidase (PTOX) genes. Color components were associated most significantly with ZEP. Our results suggest the involvement of the couple PDS/PTOX and ZEP in carotenoid accumulation, as the result of the metabolic and catabolic activities respectively. This study brings new insights in the understanding of the carotenoid pathway in non-photosynthetic organs.

## Introduction

Carotenoid compounds play an essential role in human health, preventing disease thanks to their antioxidant capacity, but also as provitamin A precursors. As humans cannot synthetize carotenoids, they have to be provided by plant-based dietary [1]. Carrot is one of the most important vegetables in the world, and a critical source of carotenoid as a large amount is accumulated in root tissues [2]. Moreover genetic resources exhibit a large range of colors and carotenoid content patterns [3], questioning the genetic control of carotenoid accumulation in carrot.

**Figure 1:**
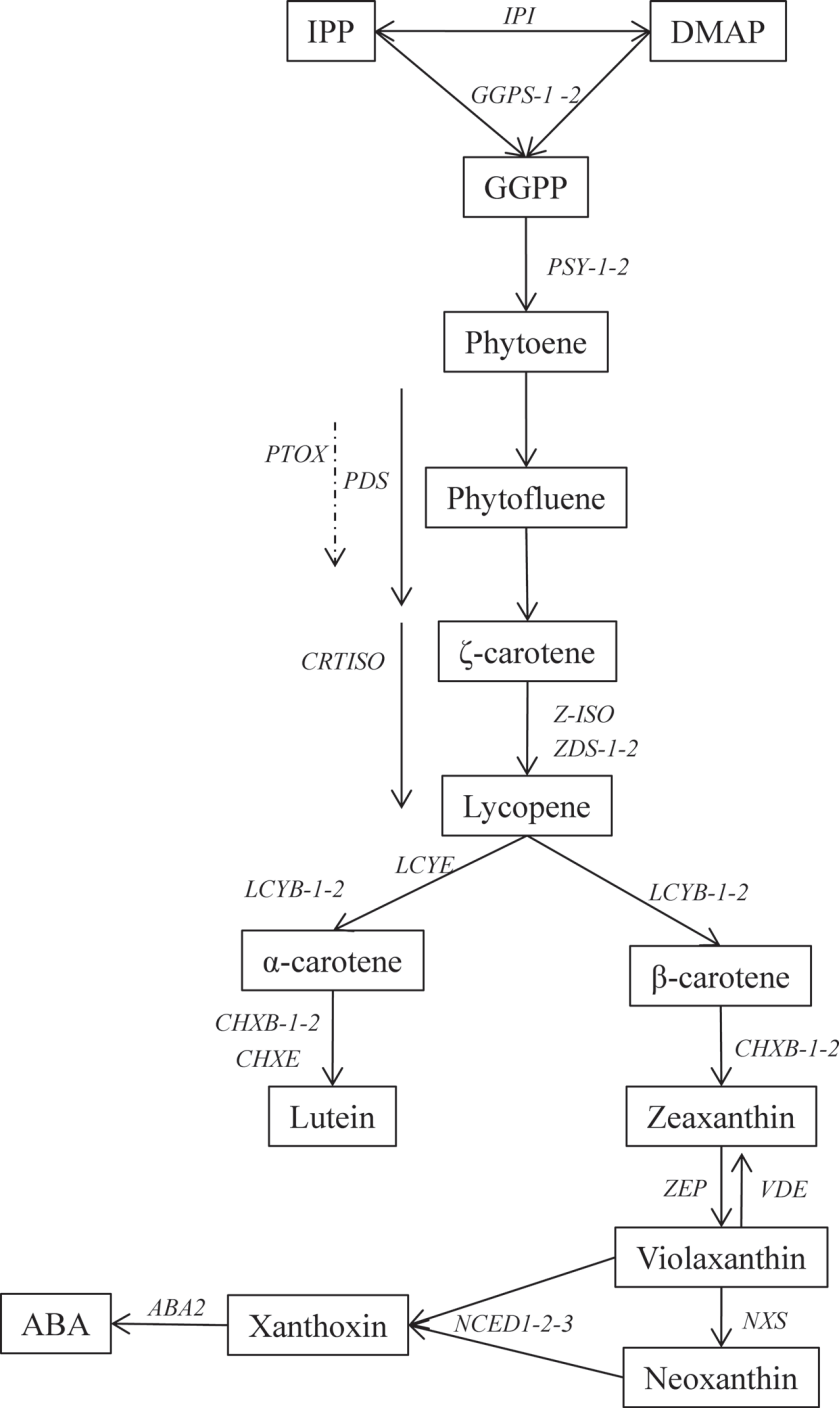
Carotenoid biosynthetic pathway in plants and gene copy number in carrot. IPP: isopentenyl diphosphate, DMAP: Dimethylallyl-diphosphate, IPI: Isopentenyl pyrophosphate isomerase, GGPS: Geranylgeranyl pyrophosphate synthase, PSY: Phytoene synthase, PDS: Phytoene desaturase, PTOX: Plastid terminal oxidase, CRTISO: Carotene isomerase, ZDS: ζ-carotene isomerase, LCYE: ε-lycopene cyclase, LCYB: β-lycopene cyclase, CHXB: β-carotene hydroxylase, CHXE: ε-carotene hydroxylase, ZEP: zeaxanthin epoxydase, VDE: violaxanthin de-epoxydase, NXS: Neoxanthin synthase, NCED: nine-*cis*-epoxycarotenoid dioxygenase, ABA: abscisic acid. Adapted from [25] and [58].

Carotenoid biosynthesis is today well established (Fig. 1) and genes encoding carotenoid enzymes have been characterized in many species [4–7]. Multiple steps in the pathway have been identified as controlling the carotenoid diversity and amount in various plant organs. Substrate availability—isopentenyl diphosphate and dimethylallyl-diphosphate—is generally considered as a limitating factor as well as the catabolic activity [4,8]. Accumulation of phytoene, controlled by the phytoene synthase and the phytoene desaturase, has emerged as a key regulatory step in the accumulation of carotenoids in various storage organs [9–13].

Many studies have shown that carotenoid biosynthetic genes are involved in the genetic control of carotenoid content (maize [14], tomato [15], wheat [12,16], pepper [17]). Depending on the species, all carotenoid biosynthetic genes may be involved in the genetic basis of carotenoid content and are therefore meaningful candidate genes [4]. In some species, engineering the pathway using biosynthetic genes is now possible for crop enhancement of the carotenoid content. Golden Rice is such an example of metabolic pathway engineering for quality enhancement [18].

However, little is known about the genetic control of carotenoid accumulation in carrot. Heritability of carotenoid content in carrot roots has been estimated by [19] and ranges from 28% to 98% depending on the compound and the investigated genetic background. Two major loci *Y* and *Y2* governing the orange intensity of xylem/phloem were identified [20]. The *Y* locus may block the synthesis of carotene and xanthophyll, whereas the *Y2* locus determines the carotene accumulation but not the xanthophyll one [21, 22]. A path analysis showed that phytoene accumulation may be one key step limiting carotenoid accumulation in white roots [9]. This was confirmed by [11], who turned a white rooted carrot in orange by overexpressing a phytoene synthase gene. Recently, a polymorphism of carotene hydroxylase CYP97A3 controlling the α-carotene content was identified [23] and the authors suggested a negative feedback regulation on PSY determining the carotenoid flux. Only two studies [21,24] have studied the genetic determinism of carotenoid content in carrot roots by linkage mapping, using a cross between an orange cultivated carrot and a white wild one. Moreover, almost all biosynthetic genes have been sequenced and mapped in carrot [25]. Two major QTLs governing carotenoid accumulation were localized, with some of carotenoid biosynthetic genes – zeaxanthin epoxydase, carotene hydroxylase and carotenoid dioxygenase families – mapped in the confidence interval or near these two QTLs.

As QTLs might be population-specific, association mapping has emerged in the last decade as an alternative to linkage analysis to dissect the basis of quantitative traits in plants. Such studies address the relationship between marker-based polymorphism and phenotypic variation in a diversified population. Using a diversified population may increase the resolution of such a study by using all ancestral recombination events [26]. One major interest of such a population is also the opportunity to study many alleles compared to a bi-parental cross study [27]. Association mapping targeting candidate genes has proven successful in many instances [28–31] and might bring new insights for carotenoid content as the genetic pathway has already been dissected through forward and reverse genetics in many organisms. However, one pitfall in association mapping is the lack of power when performed in structured panels. Structure can lead to an increase of false discovery rate. Indeed, false positives can be detected when phenotypic traits are correlated with underlying population structure at non causal loci [32]. *Daucus carota* L. genetic resources are known to be structured into two distinct genetic groups [33–35] according to their geographical origin. Moreover carotenoid content pattern is closely linked to these genetic groups: cultivars with high lycopene content belong mostly to one genetic group. In this case, association mapping can typically lead to false positive detection.

Population stratification can be estimated with different ways, and added as a covariate in association models, limiting the detection of false positive. The first proposed model [36] – called Q model – estimates the population stratification with the Bayesian model, now implemented in Structure software [37,38]. Then a principal component analysis has been proposed to correct for the structure [39]. The use of both Kinship matrix (K) and population structure in a unified mixed model approach to account for relatedness between individuals has been proposed by [27].

In order to overcome the structure bias, we have created a specific unstructured population with a broad genetic basis to perform an association mapping study for carrot root carotenoid content.

The aim of this study was to investigate the implication of biosynthetic genes in carrot root carotenoid content and related color traits using a broad unstructured population in a candidate-gene association approach. This work will offer new insights in the global understanding of the carotenoid biosynthetic pathway in carrot. This will allow us to identify favorable alleles with associated markers usable in marker-assisted selection (MAS) for product quality enhancement.

## Materials and Methods

### Plant material

The discovery population consisted of 380 individuals from the third generation of inter-crossing of an initial panel of 67 cultivars, each represented by 6 individuals. This panel represents a large diversity of cultivated carrot: three white (Europe and Middle-east), eight yellow (Europe, Central Asia, Asia), two red (Asia), 45 orange (Europe, South & North America, Australia, Madagascar, central Asia, Asia) and eight purple ones (Europe & Middle East). Pollinators were introduced at maximum blooming to avoid genetic drift and seeds were harvested from each plant in a balanced way as suggested by [40]. At each generation, 150 seeds were randomly chosen and sown for the next one. The third intercrossing generation was sown in the field in Agrocampus-Ouest (Angers, France) and grown following standard practices. Roots were harvested at 97 days after sowing. At harvesting, 380 individuals were randomly chosen.

### Phenotypic data

#### Color evaluation by spectrocolorimetry

Since color is a quality attribute and may be considered as an indirect measure of carotenoid content [41–42], color was evaluated with a CM2600d Minolta (Japan) spectrocolorimeter equipped with a 5 mm measuring area. The illuminant used was D65 and calibration was done with a white standard and specular component of light was excluded. Two measures were done for epidermis and secondary phloem and one for secondary xylem.

CIELAB color space coordinates (L*, a*, b*, C* and h) were recorded. Data represent the mean of measures per tissue type.

Then roots extremities were cut off and roots were immediately ground and frozen in liquid nitrogen and stored at −80°C. Both carotenoids and DNA were extracted from the same root sample. Dry matter was determined after drying approximately 10 grams sample at 55°C for 7 days.

#### Carotenoid quantification by HPLC

The procedure was adapted from [43]. Extraction was done on approximately 500 mg of crushed frozen material to which 50 μL of β-apo-8’-carotenal at 0.1 g/L was first added as an internal standard. Samples were mixed with 7 mL MgCO_3_ 0.57%, 3,5-di-*tert*-butyl-4-hydroxytoluene (BHT) 0.1% in methanol, then vortexed, and mixed with 7 mL of 0.1% BHT-containing chloroform. After 15 min incubation in darkness, 7 mL of ultrapure water were added, and samples were centrifuged at 236 g for 10 min. One milliliter from the lower layer was concentrated under vacuum evaporation, and the dry extract was dissolved in 200 μL of acetonitrile/dichloromethane (50:50, v/v) containing 0.1% BHT. Samples were kept at 4°C and protected from direct light during the whole procedure. Extraction was carried out in duplicates.

The analyses for carotenoid quantification were done on a Shimadzu (Shimadzu Corporation, Kyoto, Japan) HPLC equipped with a ternary pumps (LC-10AT *VP*), a thermostated autosampler (SIL-10AD *VP*), a photodiode array detector (SPD-M10A *VP*), a controller (SCL-10A *VP*), an on-line degasser (Degasys DG-1310), and a temperature controller (Crococil). Data acquisition and processing were done using the LC workstation Class-VP (Shimadzu). The procedure was adapted from [44]. Carotenoids were separated along an YMC C30 (YMC, Japan) column (150 × 4.6 mm; 3 μm) kept at 22°C. The mobile phases were MeOH/ACN/H_2_O 84/14/2 (v/v/v) as eluent A, and methylene chloride as eluent B. The elution program had the following proportions of solvent A: 0–5 min, 95–70%; 5–25 min, 70–45%; 25–30 min, 45–10%; 30–35 min, 10–95%; 35–42 min, 95%. The flow rate was 0.9 mL/min. The injection volume of filtered sample (0.45 μm PTFE membrane filter) was 20 μL. The detection was monitored from 200 nm to 800 nm.

Carotenoid compounds were identified according to their elution order and UV-visible spectrum in comparison with their authentic standards (analysed individually and in combination in the conditions used for samples), and with data from the literature [45] when unavailable. Quantification was done at 296 nm (phytoene), 348 nm (phytofluene), 450 nm (lutein, β-apo-8’-carotenal, α-carotene, β-carotene), and 472 nm (lycopene), based on internal calibration using β-apo-8’-carotenal, β-carotene calibration curve and extraction yield. Data represent the mean of two assays per individual and are expressed as β-carotene equivalents in mg per 100gr of dry mater (DM).

### Genotypic data

#### SNP discovery, haplotype-tagging SNP selection and genotyping

Assuming results from previous studies, we choose carotenoid biosynthetic genes as potential good candidate genes to explain the observed variation in root carotenoid content.

Polymorphic sequences for *IPI, PDS, LCYE, LCYB1, CHXE, ZEP* and *CRTISO* genes were obtained from previous works [33,35].

In order to identify polymorphism in other candidate genes, 48 lines representing a large diversity were sequenced for *GGPS2, ZDS1, PTOX, PSY1, PSY2, NCED1*-*2-3, LCYB2* and *ABA2* gene fragments. Primers were designed based on publicly available databases (S1 Table). PCR reactions, cycling conditions and sequencing protocol were identical as in [35]. All sequences were aligned by using Geneious software and haplotypes were inferred with DnaSP.

The sequence for a marker associated to the *Y2* locus responsible for carotenoid accumulation was obtained from [46].

#### SNP Genotyping

DNA was isolated and purified with a modified CTAB protocol [47] and DNA concentration was adjusted to 15 ng/μL. SNP genotyping was carried out by KASPar Assay (KBioscience – LGC Genomics). This technology is based on a property competitive allele-specific PCR system. The allele detection is based on a FRET quencher cassette which allows bi-allelic discrimination of known SNPs and InDels.

A total of 470 SNPs were found over all the 12,351 bp sequences from 17 genes. We identified 169 haplotypes over the 17 genes. Haplotype-tagging SNPs (HtSNPs) were chosen to maximize the number of haplotypes. We were able to design primers for 109 SNPs out of the 120 predicted (context sequences are provided in S2 Table). Finally, 93 SNPs were used after removing SNPs with Minor Allele Frequency (MAF) lower than 5% and missing data higher than 20%.

For each gene, haplotypes were reconstructed using PHASE implemented in DNASP [48]. LD within and between candidate genes was assessed with r^2^ generated by TASSEL [49].

#### SSR Genotyping

For SSR markers, 15 primers used in [35] were chosen regarding to their genome coverage and reproducibility [50]. PCR reactions, capillary electrophoresis and fragment sizing were identical as in [35].

### Population structure and relatedness

Both SSR and SNP datasets were used to investigate population stratification and relatedness between individuals as they can lead to false positive detection during association analysis.

Population stratification was first investigated on SSR dataset with the Bayesian model-based STRUCTURE software [37] which is used to infer distinct populations and to assign individuals to the identified populations. The model allowed admixture and allele correlated frequencies and was run with a burnin period of 10^5^ and a run length of 10^6^ iterations. Ten independent runs were performed for each putative cluster number (K). The range of possible K tested was from one to ten. Evanno’s method [51] was used to estimate the most probable K number.

Population stratification was also studied by PCA analysis by using SNP dataset on TASSEL software [49].

In order to study the relatedness between individuals, two Kinship matrixes were calculated by using TASSEL software based on SSR (K-SSR) markers and SNP (K-SNP) markers respectively.

### Association tests

Association analysis was performed by using TASSEL software [49]. Single polymorphism with MAF less than 5% and/or more than 20% of missing data were removed from the analysis. Marker-Trait associations were calculated using six models to evaluate the effects of population stratification and kinship: first a naive model without correction, then models correcting for population stratification estimated by STRUCTURE (Q) and by PCA (P), and finally mixed linear models taking account for relatedness between individuals estimated with SNP (K-SNP) and SSR (K-SSR) and the population structure (Q+K and P+K).

Due to multiple testing, SNPs were declared as significantly associated with a threshold of 5.3×10^−4^ *p value* as corrected with the standard Bonferonni procedure. The amount of variation explained by a SNP (r^2^) was calculated for each significant association using a simple general linear model. Haplotypes were declared significantly associated with an arbitrary threshold of 0.05 *p value* due to the small number of tested genes.

The model pertinence was evaluated by plotting *p value* in a cumulative way as described by [52]. A uniform distribution of *p values* indicates an ideal model.

## Results

### Phenotypic variation for carotenoid and color

A first visual observation of all 380 individuals showed no white or red roots. The whole population was composed with a gradient of yellow to orange roots. Due to the very low number of individuals exhibiting lycopene and limited content variability for this compound, association tests for lycopene content were not performed.

On average, β-carotene represented almost half of total carotenoid content and α-carotene represented about the third of β-carotene content (Table 1). Lutein content was relatively low as well as precursor compounds like phytoene and phytofluene. The unstructured population exhibited a large variation for all carotenoid compounds as shown by the high standard deviation.

**Table 1:** Carotenoid content variation in the unstructured population.

	**phytofluene**	**phytoene**	**lutein**	**α-carotene**	**β-carotene**	**Total**
Mean	1.436	1.134	3.624	5.900	16.388	28.831
SD	1.822	1.503	2.428	9.739	20.165	33.228
Min	0.000	0.000	0.949	0.014	0.083	1.253
Max	11.440	9.706	16.615	88.076	136.531	254.248

Traits are described by mean, standard deviation (SD), minimum (min) and maximum (max). Carotenoid contents are expressed in mg/100g of dry material. n = 380 individuals.

Color components were measured for epidermis, secondary phloem and secondary xylem. Phenotypic variations were quite similar between tissues (Table 2).

**Table 2:** Variation for color components for root epidermis, secondary phloem and secondary xylem.

**Traits**	**Epidermis**		**Secondary Phloem**		**Secondary Xylem**	
	**Mean**	**SD**	**Mean**	**SD**	**Mean**	**SD**
L*	57.46	6.92	60.23	6.73	62.00	7.913
a*	15.85	9.65	14.61	9.83	11.98	9.28
b*	36.06	7.53	39.57	6.76	38.48	7.74
C*	40.62	7.35	43.38	7.32	41.24	8.32
h	65.90	15.33	70.24	13.09	73.19	12.48

Traits are described by mean and standard deviation (SD). n = 380 individuals.

Table 3 shows the correlation between carotenoid compounds. All carotenoid compounds except lutein were highly correlated with β-carotene with a highly significant r^2^ between 0.78 and 0.99. On the contrary, lutein was not correlated with any other compound (r^2^ between 0.22 and 0.49). As secondary phloem represents the largest part of the roots, correlations between color components of this tissue and carotenoid content were investigated (Table 3). Except for lutein, all carotenoid compounds were mainly correlated with a* and h (r^2^ between 0.70 and 0.77 or 0.6 and 0.69 respectively). Lutein was not correlated with any other trait.

**Table 3:** Pearson correlation between root content of carotenoid compounds and color components of the secondary phloem.

	**phytofluene**	**phytoene**	**α-carotene**	**β-carotene**	**lutein**	**total**	L*	a*	b*	C*
phytoene	0.99***									
α-carotene	0.80***	0.78***								
β-carotene	0.90***	0.87***	0.88***							
lutein	0.49***	0.46***	0.22***	0.41***						
total	0.92***	0.89***	0.93***	0.99***	0.44***					
L*	0.45***	0.43***	0.39***	0.46***	0.06	0.44***				
a*	0.72***	0.70***	0.70***	0.77***	0.21***	0.77***	0.58***			
b*	0.07	0.06	0.06	0.08***	0.25***	0.09	0.59***	0.09		
C*	0.41***	0.4***	0.42***	0.46***	0.28***	0.47***	0.19***	0.57***	0.86***	
h	0.66***	0.64***	0.60***	0.69***	0.16**	0.67***	0.8***	0.90***	0.27***	0.23***

(Significance: ***<0.0001; **<0.001)

### Population Structure

All 15 SSR markers were polymorphic and 132 alleles were identified with a mean of 8.8 alleles per locus.

**Figure 2:**
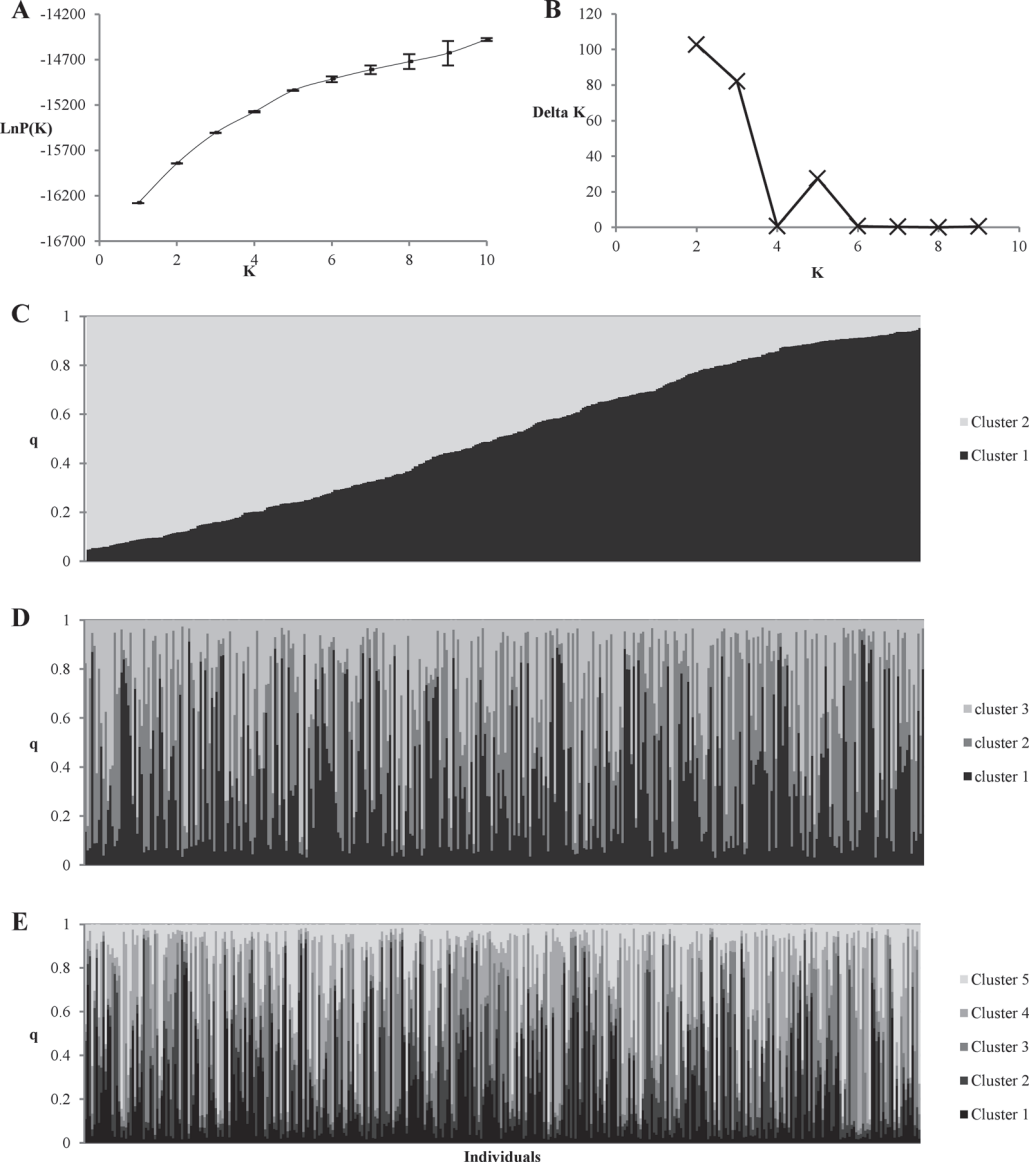
Population stratification according to STRUCTURE based on 15 SSR markers. Plots of (A) Delta K and (B) the log likelihood. Each individual is represented by a single vertical box broken in colored segments according to the number of assumed populations: 2 (C), 3 (D), 5 (E). Vertical box length is proportional to each cluster assignment probability (y-axis).

**Figure 3:**
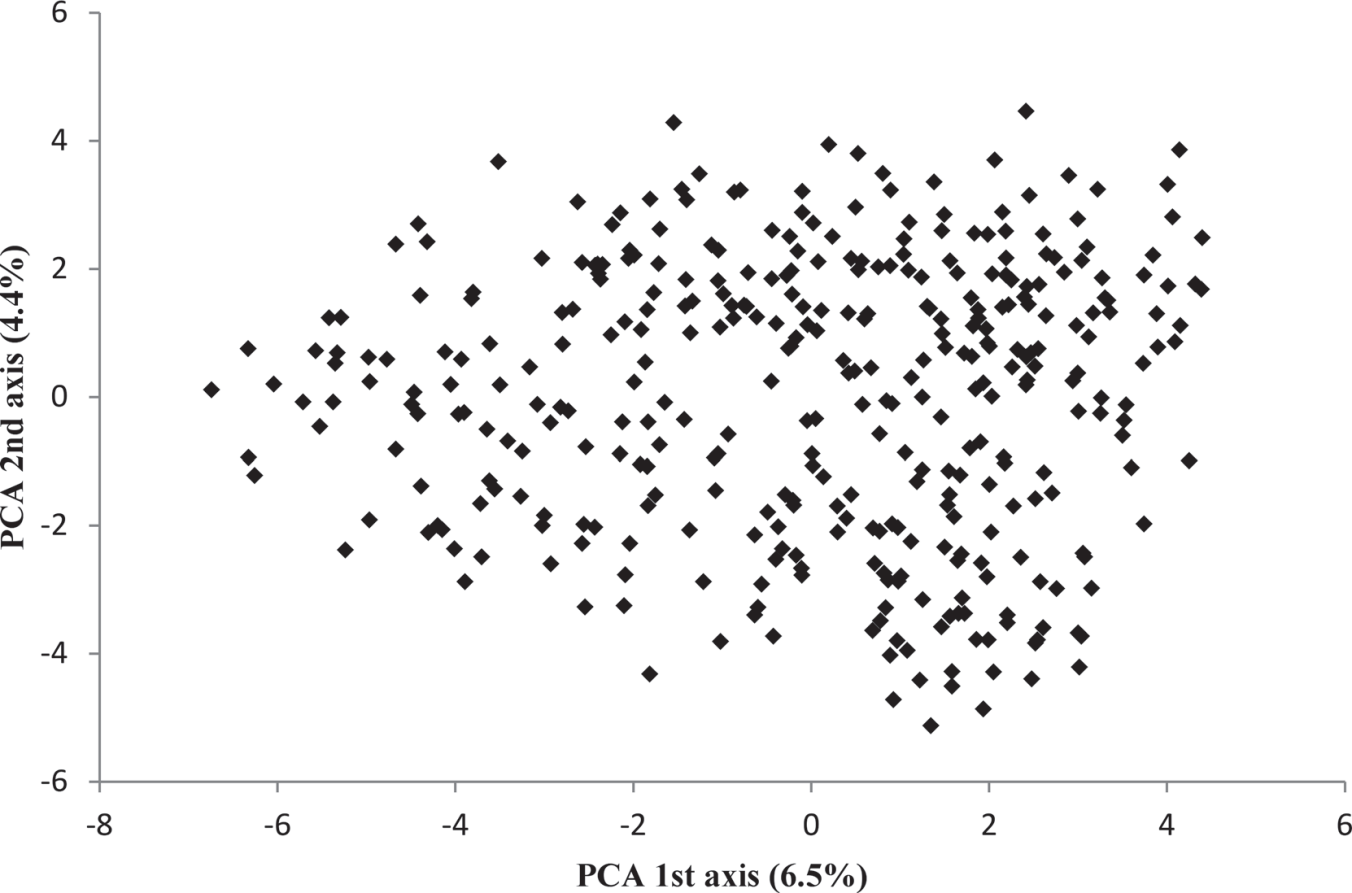
Principal component analysis of population based on SNP markers. Numbers in parentheses refer to the proportion of explained variance.

Population stratification was first investigated with STRUCTURE based on SSR markers. LnP (K) plot (Fig. 2A) did not reach a plateau as expected in the presence of structure in the sample. The number of genetic groups was also investigated with the Evanno’s method [51]. As shown on Fig. 2B, the most probable K was 2, 3 or 5. However, as shown in Fig. 2 (C, D, E) all individuals were admixed and none of them was clearly assigned to one group. At least the proportion of samples assigned to each group was roughly symmetric (∼1/K). All these elements showed an absence of structure in the population. This conclusion is reinforced by the principal component analysis performed with SNP data (Fig. 3), in which no group was clearly defined.

### Linkage Disequilibrium

No intergenic LD between genes was observed. All r^2^ between genes were less than 0.2 between SNPs located in different genes. But high or low LD was observed between SNPs within genes depending on the considered gene. *ZDS1, LCYB1, IPI, LCYE* and *ZEP* genes showed a relatively high LD between SNPs as observed by [33]. On the contrary, other genes such as *NCED* family or *PTOX* exhibited no intragenic LD (S1 Fig.).

### Association analysis

#### Model choice

**Figure 4:**
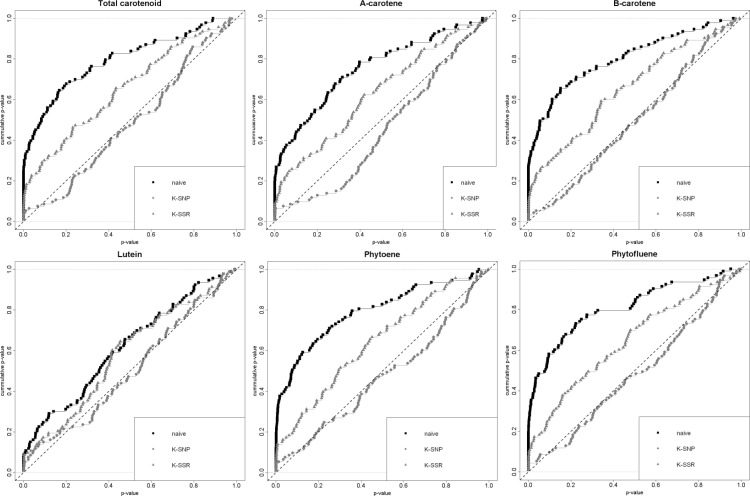
Comparison of various association mapping models for carotenoid content. Naive (black line) = Generalized Linear Model without correction. K-SSR (blue line) = Mixed Linear Model with Kinship evaluated from SSR data. K-SNP (red line) = Mixed Linear Model with Kinship evaluated from SNP data.

As Structure did not converge and PCA did not show any structure in our population, models with Q and PCA covariables are not presented. To assess the goodness of each model (naive, K-SNP or K-SSR), cumulative *p value* plots are presented in Fig. 4 for carotenoid traits and SNPs based associations. For all traits, the naive model did not perform well and an excess of low *p values* was found. At least, the K-SSR model performed better than the naive one for SNP-trait associations as well as for haplotype-trait associations. The K-SNP model showed a uniform distribution of *p values* and therefore minimized the chance of spurious association. The presented association results are thus all based on the K-SNP model. Results were similar for color components traits: the K-SNP model performed better than any other models.

#### Association results based on SNPs

**Figure 5:**
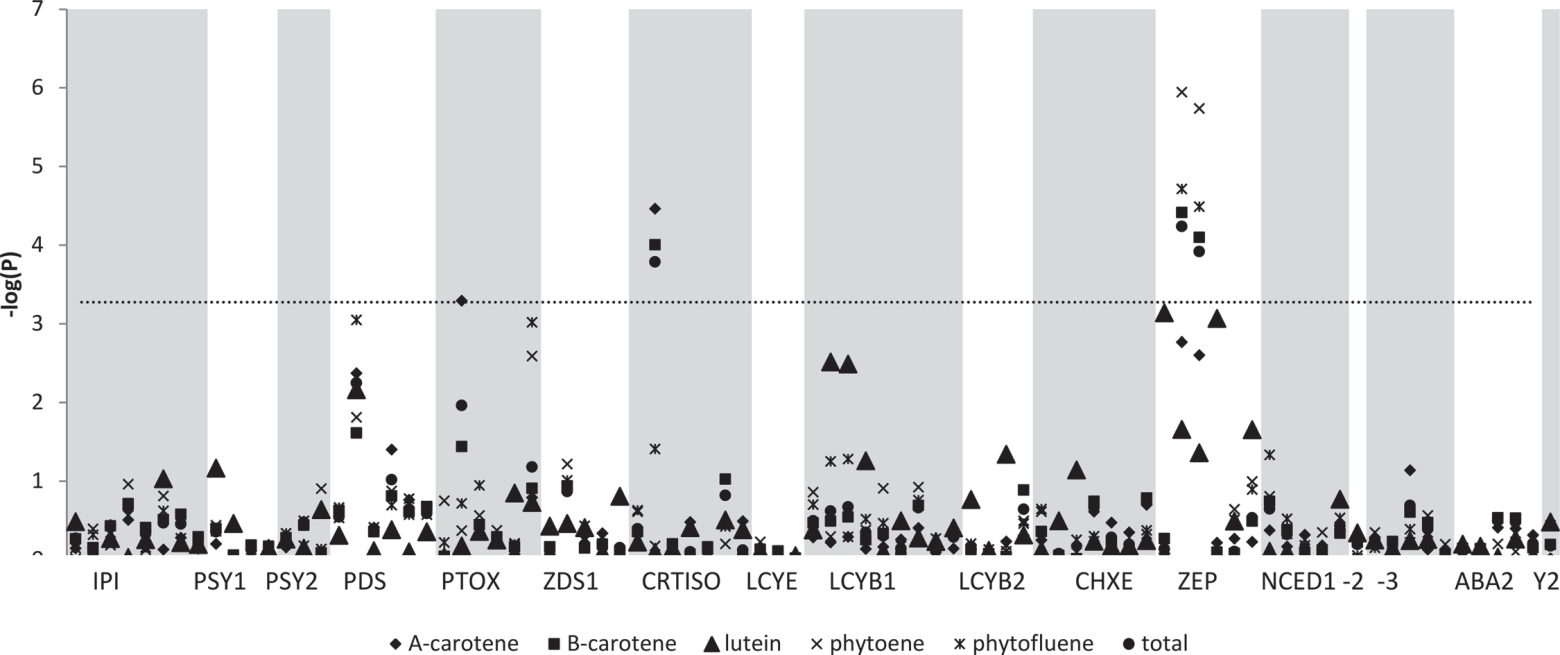
Manhattan plots of the K-SNP model for carotenoid content. Grey horizontal line indicates the significance threshold. Genes are displayed in the pathway order.

**Figure 6:**
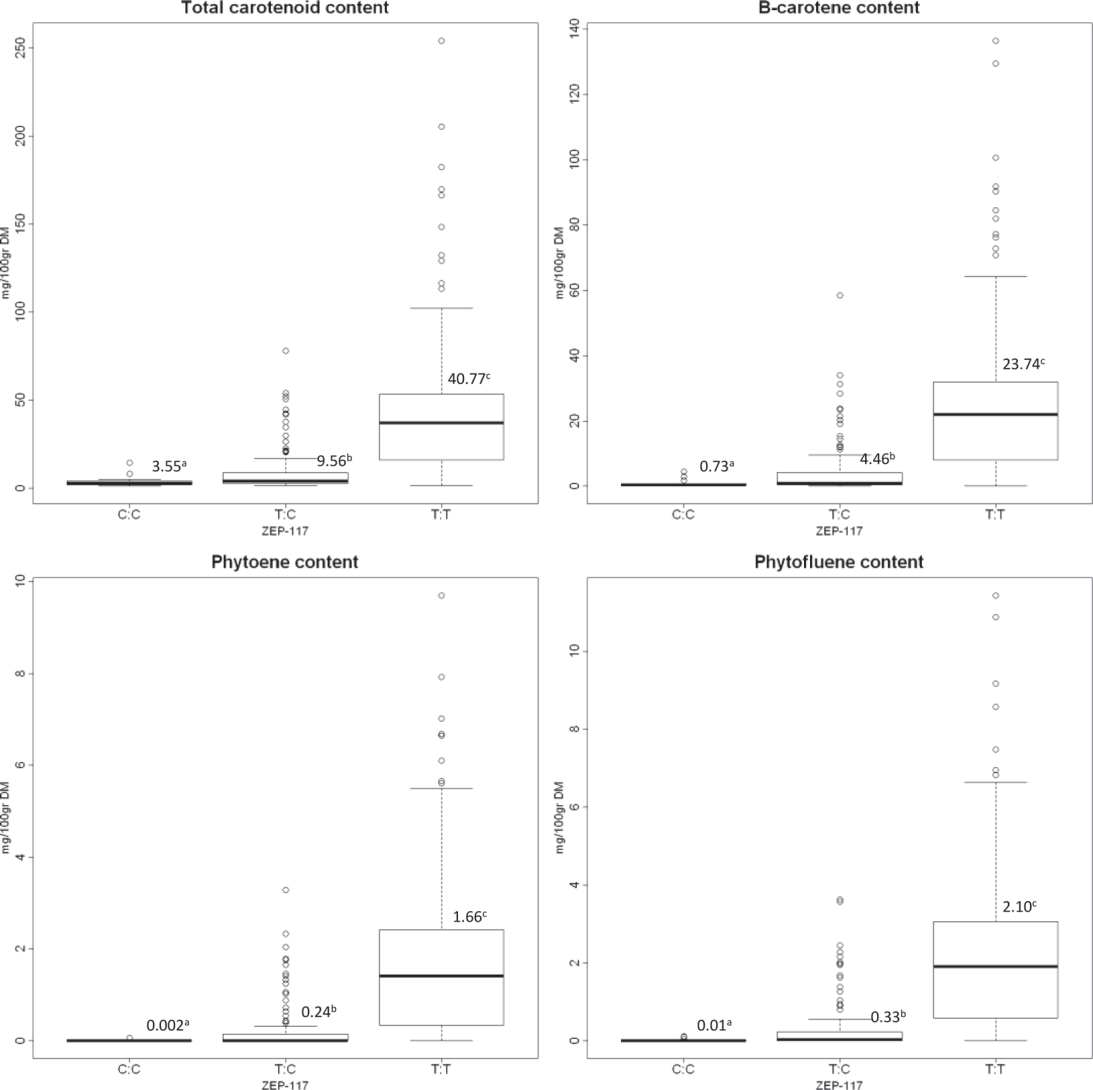
Boxplots of the ZEP-117 alleles for total carotenoids, β-carotene, phytoene and phytofluene content. Mean and group according to Kruskal-Wallis test are given for each boxplot.

With the K-SNP model, 93 SNPs were tested against 21 traits. Among the 1953 marker-trait pairs, 23 significant associations were found with a Bonferroni corrected threshold of 5,3×10^−5^ (Fig. 5). Two SNPs (ZEP-117 and ZEP-361) in the zeaxanthin epoxydase gene were associated with total carotenoids (R^2^_ZEP-117_ = 0.21), β-carotene (R^2^_ZEP-117_ = 0.22), phytoene (R^2^_ZEP-117_ = 0.22) and phytofluene (R^2^_ZEP-117_ = 0.23) content. These results were similar when carotenoid traits were expressed on a fresh matter basis (data not shown). These two SNPs were also associated with color components a* and h for all three tested tissues (Table 4). These two SNPs were in high LD (r^2^ = 1) and therefore redundant. These SNPs were located in a non-coding region. Fig. 6 shows the distribution of carotenoid content for each allele of the ZEP-117 SNP (Results were similar for the ZEP-361 SNP, data not shown). For all associated compounds, C:C, C:T and T:T genotype means were significantly different from each other (*p* < 0.05, Kruskal-Wallis test). This reveals a typical dominant action for this locus.

**Table 4:** Significant SNP-trait associations according to the K-SNP model for color components.

**SNP**	**Tissue**	**Trait**	***p value***
ZEP-117	Epidermis	a*	2.70E-06
	Epidermis	h	1.41E-05
	Secondary Phloem	a*	1.79E-06
	Secondary Phloem	h	4.00E-06
	Secondary Xylem	a*	3.32E-06
	Secondary Xylem	h	3.64E-05
			
ZEP-361	Epidermis	a*	1.46E-05
	Secondary Phloem	a*	8.11E-06
	Secondary Phloem	h	2.88E-05
	Secondary Xylem	a*	5.87E-06
	Secondary Xylem	h	3.62E-05

One polymorphism in the carotenoid isomerase gene (*CRTISO*) was associated with total carotenoids, β-carotene and α-carotene. At least one SNP in the plastid terminal oxidase (*PTOX*) – a cofactor of the phytoene desaturase – was associated with α-carotene (Fig. 5). No association was detected between the Y2 related marker and carotenoid content.

#### Association results based on haplotypes

When testing haplotypes against carotenoid content, we detected 32 associations with a *p value* lower than 0.05 (Table 5). Among these 32 significant associations, 27 were associated with three genes. Total carotenoids, β-carotene, phytoene and phytofluene content were associated with *ZEP* gene. This gene was also associated with color components for the three investigated tissues. Phytoene desaturase gene was associated with lutein, phytoene, phytofluene and total carotenoid content and with the color saturation C* of inner root. The plastid terminal oxidase gene (*PTOX*) was associated with α-carotene, phytoene, phytofluene and total carotenoid content. It was also associated with the color components b* for both epidermis and secondary phloem and C* for epidermis. At last *PSY2, ZDS1* and *NCED1* were associated with color components and *PSY1* with lutein content.

**Table 5:** Significant haplotype-trait associations according to the K-SNP model for carotenoid content and color components.

**Gene**	**Tissue**	**Trait**	***p value***
*PSY1*	whole root	lutein	0.019
*PSY2*	Secondary Phloem	L*	0.030
		b*	0.037
			
*ZDS1*	Epidermis	h	0.027
*PDS*	whole root	lutein	0
		phytoene	0.016
		phytofluene	0.014
	Secondary Phloem	C*	0.002
	Secondary Xylem	C*	0.015
			
*PTOX*	whole root	α-carotene	0.002
		phytoene	0.008
		phytofluene	0.017
		total	0.016
	Epidermis	b*	0.014
		C*	0.028
	Secondary Phloem	b*	0.009
			
*ZEP*	whole root	β-carotene	0.030
		phytoene	0.049
		phytofluene	0.050
		total	0.042
	Epidermis	L*	0.028
		a*	0.004
		b*	0.032
		C*	0.023
		h	0.005
	Secondary Phloem	L*	0.003
		a*	0.004
		b*	0.046
		h	0.001
	Secondary Xylem	a*	0.004
		h	0.016
			
*NCED1*	Secondary Xylem	L*	0.033

## Discussion

### Unstructured population and consequences for association study

Carrot genetic resources often exhibit a strong stratification [33–35] which can lead to false positive detection when association mapping is performed. Here we conducted a candidate gene association study on root carotenoid content by using an unstructured population. Both SNPs and SSRs confirmed the absence of population stratification in this population. The same set of SSR markers were used in [35] and revealed a strong population stratification on a diversified panel of carrot accessions. So neither Q matrix nor PCA component were used in association mapping model. But the naive model showed an excess of low *p values* and was therefore not fitting. As all individuals descended from the same parents, relatedness between individuals was relatively high and association model had to be corrected for relatedness. A kinship matrix was still needed.

Auzanneau et al. [53] already demonstrated in ray grass the interest of using a panmictic population in association mapping studies to overcome the population stratification bias. The choice of parents appeared to be essential. In our original set of cultivars set, red rooted carrot was under represented and we were not able to observe red roots after three generations. As population stratification was disrupted after three intercross generations, the choice of the parents should be based more on the phenotypical variation representativity than on original stratification. In order to reach a large variation for the targeted traits after several intercross generations, the first generation must exhibit a large range of phenotypic variation but also an equilibrated representation of phenotypic patterns. Indeed, as mentioned by [53], detected associations may be different depending on population parents.

### Major role of the catabolic gene zeaxanthin epoxydase

Zeaxanthin epoxydase gene polymorphism was associated with total carotenoid, β-carotene, phytoene and phytofluene content. Moreover, associations with color components a* and h were also detected. As the two polymorphisms associated with traits were in a non-coding region, we were unable to detect the causal polymorphism of the phenotypic variation. But as this gene exhibited a moderate LD as shown in S1 Fig. and in [33], the causal polymorphism may be located somewhere else in the LD block.

*ZEP* is one of the major steps in the carotenoid pathway. Nevertheless, carotenoid accumulation in various organs is known to be the result of biosynthesis, degradation and storage [4,8]. An impaired function of the zeaxanthin epoxydase protein may result in an accumulation of β-carotene. As β-carotene, phytoene and phytofluene were highly and positively correlated, a high level of β-carotene was often associated with a high level of precursors phytoene and phytofluene. High level of phytoene and phytofluene may also be the result of a large β-carotene accumulation due to a reduced degradation. It also seems that the zeaxanthin epoxydase gene may drive the biosynthesis pathway towards the β-branch.

In a previous study, [21] performed a QTL detection in a biparental cross between a wild and an orange cultivated carrot. One major QTL was detected for α- and β-carotene, phytoene, zeta-carotene, phytoene and total carotenoid content but not for lutein content. As the marker Y for the *Y2* locus related to carotenoid accumulation was mapped in the confidence interval of this QTL and the STS (Sequence-Tagged Site) marker *ZEP* was mapped outside of the confidence interval, the authors concluded that the role of *ZEP* was not obvious to explain the *Y2* locus. According to our results, we suggest that ZEP may one probable candidate gene underlying the *Y2* locus.

Previous results on potato tubers also showed an association between *ZEP* and flesh color [54]. A specific allele only present in orange colored flesh genotypes was identified. This allele showed a reduced level of expression probably due to a large retrotransposon in the first intron. Similar observations were done in maize. Carotenoid accumulation in kernel was inversely associated with *ZEP* transcript levels [55]. However, no significant variation of *ZEP* transcript abundance between cultivars was found during carrot root development [43]. This suggests that a putative transcription difference would be at the allele level, which needs to be confirmed by a further study on *ZEP* alleles.

In tomato, the mutation *high pigment 3* (*hp3*) occurred in the *ZEP* gene, leading to a 30% increase of carotenoid accumulation in the mature fruit [56]. These results, with related effects on ABA content and carotenoid storage capacity, reinforce our conclusion that *ZEP* is a major candidate gene governing carotenoid content in carrot roots.

### Limitating role of the synthesis phytoene desaturase gene

We identified *PDS* and his cofactor *PTOX* [57] as associated with total carotenoid, phytoene, phytofluene, α-carotene and lutein content. This suggests that these genes are involved in the global carotenoid accumulation. An early regulation of the pathway may explain the large number of associations detected for these genes. These results are consistent with previous results which identified phytoene accumulation as a major regulatory step in the carotenoid pathway in plants [4,6].

Moreover a putative signature of selection for *PDS* after domestication of carrot was shown by [58]. This may be in relation with an early control of the metabolic pathway. *PTOX* has been identified as playing a major role in carotenoid accumulation. *Arabidopsis* mutant IMMUTANS [59,60] and tomato mutant *ghost* [61] exhibit an impaired function of PTOX leading to a deficient phytoene desaturation and an accumulation of the carotenoid precursor phytoene.

Just et al. [21] also identified *CHXE, NCED2* and *PDS* as potential candidate genes linked to another major carotenoid QTL, proposed as the *Y* locus. But they could not conclude on the effect of each gene in this QTL region. As neither SNPs nor haplotypes from *CHXE* and *NCED2* were associated with carotenoid traits, our results suggest that *PDS* may be one probable candidate explaining the *Y* locus.

Unfortunately, *PTOX* is not yet mapped into the carrot genome, which would be needed to better explain its role. A recent study suggests a complicated role for the PTOX which is also involved in chloroplast biogenesis and in photosystem II photoprotection [62].

### Towards a particular mechanism driving the metabolic flux through the α-branch to lutein

The orientation of the pathway towards the α-branch or the β-branch results in different pattern of carotenoid content. However, the underlying mechanism remains unclear in carrot. The metabolic node is known to be a major regulation step in the pathway [4]. For example, variation in *LCYE* in maize explained 58% of the variation in the two branches [63]. This was also observed in *Arabidopsis thaliana* [64], *Brassica napus* [65] and *Solanum tuberosum* [66]. But we did not detect any association for α-carotene and lutein content with the ε-lycopene cyclase gene. This suggests a more complex regulation orientating the pathway to one of the branch, controlled by a genetic factor different from the tested carotenoid biosynthetic genes. Actually, Arango et al. [23] have shown the specific role of carotene hydroxylase *CYP97A3* gene in the α-carotene level.

The study of correlation between all traits showed the independence of lutein content. All carotenoid compounds except lutein were correlated together. This also suggests a particular mechanism in lutein accumulation from α-carotene.

## Conclusion

For the first time, we performed an association analysis on *Daucus carota* L. Moreover we developed an original unstructured population with a broad genetic basis, limiting the risk of spurious association. However, as all individuals descended from the same parents, relatedness estimated with kinship matrixes had to be included in the association model.

We identified several SNPs and genes associated with carotenoid content and color components. Our results bring evidence that zeaxanthin epoxydase and phytoene desaturase are candidate genes involved in carotenoid accumulation of non-photosynthetic organs. Our study brings new insight into the carotenoid pathway functioning by stressing out two major steps in carotenoid metabolism and catabolism in a storage organ. Functional validation and dissection of the regulation of *ZEP* expression may clarify the mechanisms involved in carotenoid accumulation. Clarification of the involvement of the *PTOX* has also to be investigated. A mechanism explaining both the accumulation of xanthophylls and the pathway orientation towards the α-branch as well as lutein accumulation still remains to be specified. However, the genes identified in this study as associated with color components and carotenoid content may be useful in marker-assisted selection for carotenoid content enhancement in a breeding program.

## Supporting Information

S1 Table Primers used to amplify candidate genes fragments.
(DOC)

S2 Table Context sequences of genotyped SNPs.
(XLSX)

S1 Fig Linkage disequilibrium between SNPs in all genes. r^2^ and LD significance are represented at the top and bottom of the matrix, respectively.
(TIF)
