# Supplementary material for: Carotenoid Content and Root Color of Cultivated Carrot: A Candidate-Gene Association Study Using an Original Broad Unstructured Population
Source: PLoS One. 2015 Jan 23;10(1):e0116674. doi: 10.1371/journal.pone.0116674 (PMC4304819; doi:10.1371/journal.pone.0116674)
Supplement: S1 Table — Primers used to amplify candidate genes fragments. (DOC) [file pone.0116674.s001.doc]

**Table S1. Primer used to amplify candidate genes fragments.**

| Gene | Accession number | Primer F | Primer R | Amplicon size (bp) | |
| --- | --- | --- | --- | --- | --- |
| *GGPS2* | DQ192185* | | AGCCCATGAAAACTCACTCTTGTCCA | ACCGCATCGCTTCGTGGATGA | 350 |
| *PSY1* | DQ192186* | | ACCAGAGCGCCGAAGAGCTG | TCTCTGTGGTGGCCTTTGATTCC | 850 |
| *PSY2* | DQ192187* | | GTGGTGCAGAAGAACTGATGAATTG | AGCAAGCCCTAAAGCCAAAGCA | 1000 |
| *ZDS1* | DQ222430* | | GTAGCCCTTGCCCTTAGTCCA | GGCAATGCCTGAAATGTATGTCTGT | 600 |
| *PTOX* | JG756246-JG764798-JG760793 | | GGTTGGTGGAGAAGGGCTGACT | AAGCGATCAAACCACCAAGCA | 1000 |
| *LCYB2* | AF208530 | | GTGGATGAATTTGAGGCTATGGGG | CTCTCCAATCCATAAGCACCATTC | 450 |
| *NCED1* | DQ192200* | | TTCGCAACGGAGCCAATCCG | CCAACCCCGCATTCGCAACC | 350 |
| *NCED2* | DQ192201* | | ACGGGGCCAACCCCCATTTG | AATTTTGGGGTGAGCAATCATCGTG | 500 |
| *NCED3* | DQ192202* | | CCAGTCCAGCACCACCTCCCT | AGGCGATCCACCTTGTATCATTTCT | 800 |
| *ABA2* | JG770083 | | AGCTCGCATAATGATCCCTCGGA | GTGATTCACACATGTAACACCTCCA | 450 |

*Sequences obtained from Root & Bulb Genomics Database (RoBusT)
