# Supplementary figures and images for: Carotenoid Content and Root Color of Cultivated Carrot: A Candidate-Gene Association Study Using an Original Broad Unstructured Population
Source: PLoS One. 2015 Jan 23;10(1):e0116674. doi: 10.1371/journal.pone.0116674 (PMC4304819; doi:10.1371/journal.pone.0116674)

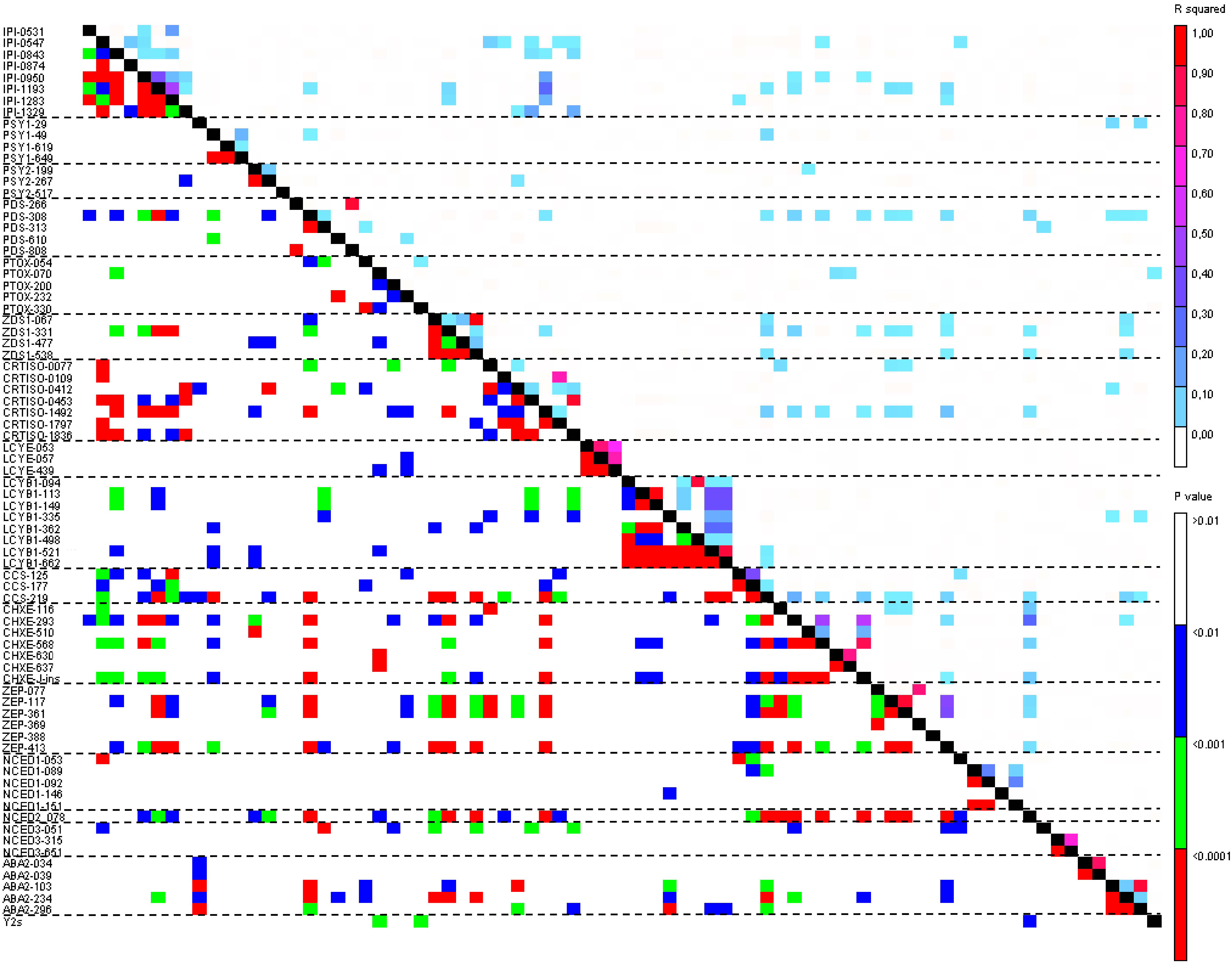

Supplement: S1 Fig — r2 and LD significance are represented at the top and bottom of the matrix, respectively. (TIF) [file pone.0116674.s003.tif]
